# TRAF5 Is a Downstream Target of MAVS in Antiviral Innate Immune Signaling

**DOI:** 10.1371/journal.pone.0009172

**Published:** 2010-02-11

**Authors:** Eric D. Tang, Cun-Yu Wang

**Affiliations:** Laboratory of Molecular Signalling, Division of Oral Biology and Medicine, University of California Los Angeles School of Dentistry, Los Angeles, California, United States of America; New York University, United States of America

## Abstract

The recognition of nucleic acids by the innate immune system during viral infection results in the production of type I interferons and the activation of antiviral immune responses. The RNA helicases RIG-I and MDA-5 recognize distinct types of cytosolic RNA species and signal through the mitochondrial protein MAVS to stimulate the phosphorylation and activation of the transcription factors IRF3 and IRF7, thereby inducing type I interferon expression. Alternatively, the activation of NF-κB leads to proinflammatory cytokine production. The function of MAVS is dependent on both its C-terminal transmembrane (TM) domain and N-terminal caspase recruitment domain (CARD). The TM domain mediates MAVS dimerization in response to viral RNA, allowing the CARD to bind to and activate the downstream effector TRAF3. Notably, dimerization of the MAVS CARD alone is sufficient to activate IRF3, IRF7, and NF-κB. However, TRAF3-deficient cells display only a partial reduction in interferon production in response to RNA virus infection and are not defective in NF-κB activation. Here we find that the related ubiquitin ligase TRAF5 is a downstream target of MAVS that mediates both IRF3 and NF-κB activation. The TM domain of MAVS allows it to dimerize and thereby associate with TRAF5 and induce its ubiquitination in a CARD-dependent manner. Also, NEMO is recruited to the dimerized MAVS CARD domain in a TRAF3 and TRAF5-dependent manner. Thus, our findings reveal a possible function for TRAF5 in mediating the activation of IRF3 and NF-κB downstream of MAVS through the recruitment of NEMO. TRAF5 may be a key molecule in the innate response against viral infection.

## Introduction

The innate immune system is the first line of host defense, regulating infection directly and signaling to the adaptive immune system [1], [2]. The enhanced production of type I interferons, including beta interferon (IFN-β), is an early and critical component of the innate immune host response to virus infection [3]. Type I interferon production can be stimulated by germ-line encoded pattern recognition receptors (PRRs) including those belonging the Toll-like receptor (TLR) and RIG-I-like receptor (RLR) families [4]. TLRs comprise a family of PRRs that can recognize pathogen-associated molecular patterns (PAMPs) including lipopolysaccharides (LPS), lipoproteins, single-stranded RNA (ssRNA), double stranded RNA (dsRNA), and unmethylated CpG-containing DNA. Cytosolic RNAs are recognized by members of the RLR family including the RIG-I and MDA-5 RNA helicases.

Biochemical and genetic studies suggest that RIG-I and MDA-5 have nonredundant roles as cytoplasmic receptors for different types of viral RNA-derived PAMPs [5], [6], [7], [8], [9]. Different RNA species are recognized by either RIG-I, MDA-5, or a combination of both depending on the types of RNA-derived PAMPs that are displayed. RIG-I has been found to recognize preferentially 5′-triphosphate poly-U/A-rich single-stranded RNA and short double-stranded (ds) RNA [10], [11], [12], whereas MDA-5 recognizes long dsRNA [13]. RNA species that are recognized by RIG-I can also be derived from the transcriptional products of RNA polymerase III using AT-rich double-stranded DNA as a template [14], [15]. Preferred RIG-I RNA ligands are able to form a stable complex with RIG-I and stimulate its ATPase activity, thus altering its conformation to an “active” signaling form [16], [17]. The final “active” form of RIG-I is thought to be one in which the two N-terminal caspase recruitment domains (CARDs) are exposed to allow for homodimerization and binding to MAVS.

MAVS function is dependent on both its C-terminal transmembrane (TM) domain and its N-terminal caspase recruitment domain (CARD). The TM domain allows MAVS to insert in the outer mitochondrial membrane and permits its dimerization in response to cytosolic RNA. Dimerization of the N-terminal CARD domain of MAVS allows it to associate with and activate the downstream signaling molecule and ubiquitin ligase TRAF3. TRAF3 mediates the activation of the transcription factor IRF3 and IRF7 through an unclear mechanism. However, although TRAF3 −/− MEFs display defective innate immunity to virus, they only reveal a partial defect in IFN-β production and enhanced activation production of inflammatory cytokines [18]. Thus, other downstream targets for MAVS other than TRAF3 likely exist.

Several other proteins have been implicated to play a role in RLR signaling downstream of MAVS. Recently a molecule called MITA/STING has been identified as an important mediator of type I interferon production by cytosolic nucleic acids [19], [20]. While MITA/STING plays a critical role in the cellular response to many forms of cytosolic DNA, it appears to largely augment signaling pathways involving RIG-I and MAVS [19], [21]. Several other molecules have also been implicated to play roles as adaptor proteins downstream of MAVS and TRAF3 including NEMO, TANK, SINTBAD, and NAP1 [22], [23], [24]. NEMO is an essential regulatory subunit of the canonical IκB kinase (IKK) complex and has been implicated to play a role in both IRF3/7 and NF-κB activation downstream of MAVS[24]. TANK, SINTBAD, and NAP1 are related proteins that share a defined TBK1-binding sequence that can be used to directly bind to TBK1 [22]. Upon virus infection, TANK has been shown to associate with MAVS as well as TRAF3 and TBK1 [23]. Also, TANK is able to bridge an interaction between NEMO and TBK1 [24]. However, how NEMO might be recruited to the MAVS signaling complex remains unclear.

Here we have uncovered an important role for TRAF5 in RLR signaling. We find that the dimerization of MAVS allows it to associate with TRAF5 and induce its ubiquitination. In addition, TRAF3 and TRAF5 both mediate the recruitment of NEMO to the MAVS signaling complex. Whereas TRAF3 mediates IRF3 activation, we find that TRAF5 is important for the activation of NF-κB as well as IRF3. Our data provide novel insight into how MAVS functions in antiviral innate immune signaling.

## Results

### Dimerization of MAVS CARD Is Sufficient to Activate NF-κB

**Figure 1:**
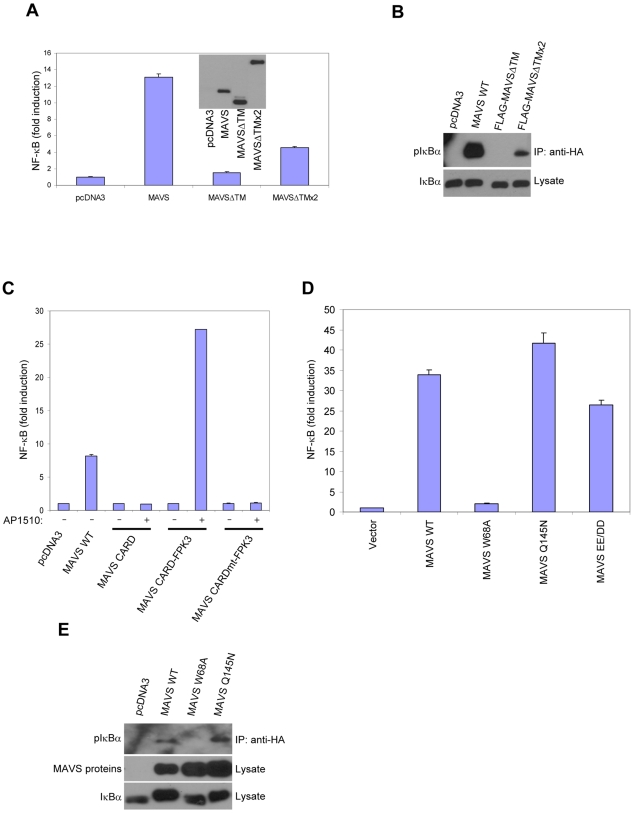
Dimerization of the MAVS CARD restores activation of NF-κB. A, C, D HEK293T cells were transfected with wild-type or mutant MAVS, and an NF-κB reporter, pLuc-PRD(II)_2_. MAVS CARDmt contains a substitution of alanine for tryptophan at residue 68. Lysates were prepared and luciferase reporter assay was performed 24 hrs later. AP1510 was added 12 hrs prior to cell lysis (C). In the inset figure in (A), a western blot of lysates for FLAG-MAVS proteins was performed (A). B, E HEK293T cells were cotransfected with HA-IkBα and pcDNA3 or MAVS proteins and lysates were prepared. Anti-HA immunoprecipitates were probed for phosphorylated IkBα. Lysates were probed for HA-IkBα and FLAG-tagged MAVS proteins (E).

Previously we found that removal of the MAVS TM domain impairs the ability of MAVS to dimerize, to activate IRF3/7 and NF-κB, and to induce IFN-β [25]. Importantly, enforced dimerization of a MAVS deletion mutant lacking its TM domain (MAVSΔTM) can restore its ability to activate IRF3/7 and induce IFN-β. These results suggested that the ability of MAVS to activate IRF3/7 is dependent on its ability to dimerize via the TM domain. To see whether the ability of MAVS to activate NF-κB was also reliant on its ability to dimerize, we examined whether enforced dimerization of MAVSΔTM could restore its ability to activate NF-κB. We tested the ability of a tandem repeat of MAVSΔTM with two copies fused end-to-end (MAVSΔTMx2) to activate NF-κB. In contrast to MAVSΔTM, the MAVSΔTMx2 fusion can phosphorylate IRF3 and activate IRF3/7 [25]. Similarly, we found that the MAVSΔTMx2 fusion, but not MAVSΔTM could elicit NF-κB activation and IkBα phosphorylation (Figure 1A and 1B). In addition, we also examined a fusion of the MAVS CARD to the three tandemly repeated dimerization domains of FPK (MAVS CARD-FPK3). Proteins attached to FPK can be induced to dimerize by the addition of the cell-permeable artificial ligand AP1510 [26]. We previously found that MAVS CARD-FPK3 could activate IRF3/7 activation and IFN-β production only in the presence of the dimerizer AP1510. We also found that this fusion protein could activate NF-κB only in the presence of dimerizer (Figure 1C). Substitution of a conserved tryptophan residue for alanine in the CARD domain (W68A), a mutation that prevents full-length MAVS from inducing IFN-β and activating IRF3 [25], also prevented MAVS CARD-FPK3 from activating NF-κB (Figure 1C).

Previously, we found using point mutants of MAVS that the CARD but not the TRAF2/3-interaction motif (TIM) was important for IRF3/7 and IFN-β induction. We examined whether NF-κB activation by MAVS was dependent on the TIM. Introduction of a mutation in the CARD (W68A) but not the TIM (Q145N) of MAVS impaired its ability to activate NF-κB and induce IkBα phosphorylation (Figure 1D and 1E). Thus, the CARD but not the TIM is required for NF-κB activation by MAVS. These results together suggest that MAVS dimerization is important for the activation of NF-κB in a CARD-dependent manner, as previously shown for IRF3/7 [25].

### TRAF5 Is an Activator of IRF3 and Interferon Production

**Figure 2:**
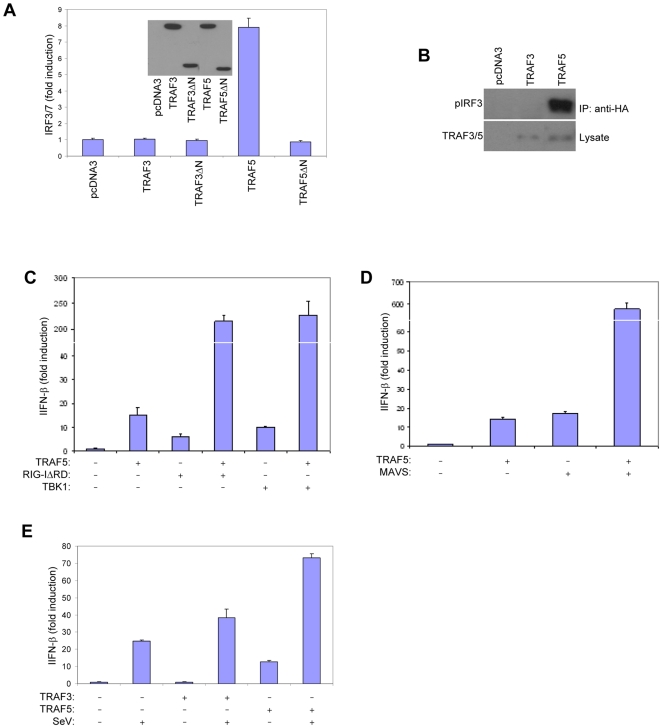
TRAF5 activates IRF3 and stimulates interferon-β production. A Luciferase reporter assay was performed in HEK293T cells cotransfected with FLAG-tagged wild-type or trunctated TRAF3 or TRAF5 expression constructs along with pLuc-PRD(III-I)_3_. In the inset figure, lysates were immunoblotted for FLAG-tagged proteins. Luciferase activity was measured 24 hrs following plasmid transfection. B HEK293T cells were transfected with FLAG-tagged TRAF3 or TRAF5 and HA-IRF3. Lysates were prepared and anti-HA immunoprecipitates were probed for phosphorylated IRF3 (Ser 396). Lysates were probed for FLAG-TRAF3 and FLAG-TRAF5. C, D, E Luciferase reporter assay was performed in HEK293T cells cotransfected with expression constructs for the indicated proteins along with pLuc-IFNβ. SeV infection was performed six hours following transfection and 18 hrs prior to luciferase assay (E). Discontinuity in graph indicates a broken Y-axis (C, D).

Although TRAF3 −/− MEFs display defective innate immunity to virus, they reveal only a partial defect in IRF3 activation and IFN-β production [27], [28]. Furthermore, these cells do not exhibit a defect in NF-κB activation, but rather display enhanced signaling mediated by increased levels of NIK [29]. Given these data and our finding that the MAVS CARD is required for both IRF3/7 and NF-κB activation, it is likely that there exists a downstream target for the MAVS CARD other than TRAF3. We chose to examine the closely related TRAF family member TRAF5. TRAF5 has been shown to be a potent NF-κB activator when overexpressed [30] but a possible role in IRF3/7 signaling has yet to be examined. We found that ectopic TRAF5, but not TRAF3 expression could stimulate IRF3 phosphorylation and activate an IRF3/7-responsive reporter suggesting that levels of TRAF5 but not TRAF3 were rate-limiting for signaling to IRF3/7 (Figures 2A and 2B). The activation of IRF3/7 by TRAF5 was dependent on the N-terminus, which contains a RING finger and multiple zinc fingers (Figure 2A). TRAF5 overexpression also activated the *Ifnb* promoter alone or in synergy with RIG-I, MAVS, TBK1, or Sendai Virus (SeV) infection (Figures 2C, 2D, and 2E). Thus, TRAF5 is an upstream activator of IRF3/7 as well as NF-κB.

### TRAF5 Associates with the Dimerized MAVS CARD

**Figure 3:**
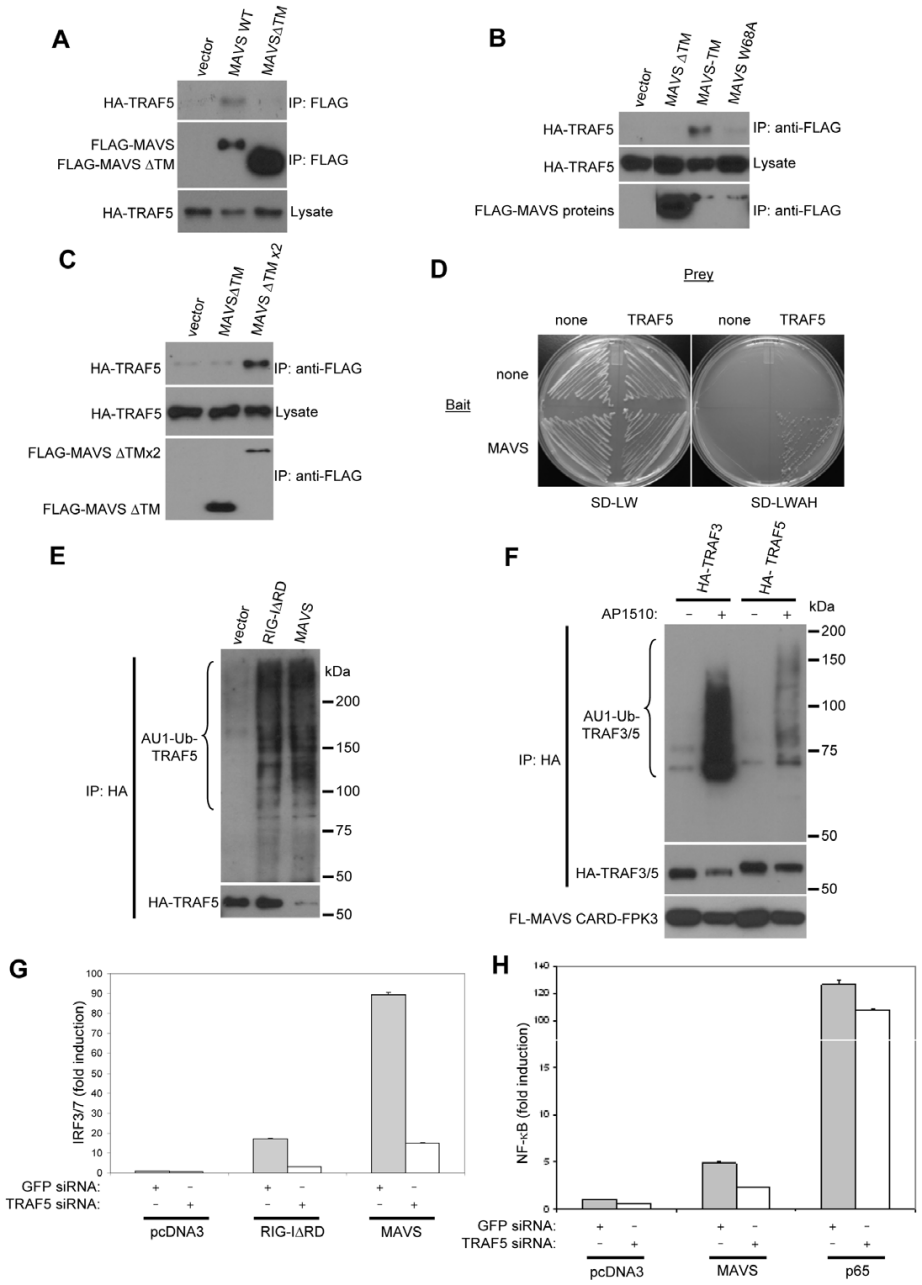
TRAF5 associates with the dimerized MAVS CARD. A, B, C HEK293T cells were cotransfected with FLAG-tagged MAVS constructs together with HA-tagged TRAF5 and whole cell lysates were prepared. Anti-FLAG immunoprecipitates were probed for HA-TRAF5 or FLAG-MAVS. Lysates were immunoblotted for HA-TRAF5. D Yeast were transformed with the bait or prey constructs indicated as described in the Materials and Methods section and streaked onto minimal SD media lacking leucine and tryptophan (SD-LW) or leucine, tryptophan, adenine, and histidine (SD-LWAH). E HEK293T cells were transfected with FLAG-tagged RIG-I or MAVS constructs, HA-TRAF5, and AU1-Ub. Whole cell lysates were prepared and anti-HA immunoprecipitates were probed for Ub-conjugated TRAF3 and TRAF5 proteins using AU1 antibody from or total protein using HA antibody. F HEK293T cells were transfected with FLAG-MAVS CARD-FPK3, HA-TRAF3 or HA-TRAF5, and AU1-Ub. Anti-HA immunoprecipitates were probed for AU1-Ub conjugates or HA-TRAF3/5. Lysates were immunoblotted for FLAG-MAVS CARD-FPK3. AP1510 was added 12 hrs prior to cell lysis. G, H HEK293T cells transfected initially with siRNAs targeting GFP or TRAF5. 48 hrs later, cells were transfected with FLAG-RIG-IΔRD, FLAG-MAVS, or FLAG-p65, and pLuc-PRD(III-I)_3_ (IRF3/7) (G) or pLuc-PRD(II)_2_ (NF-κB) (H). Luciferase activity was measured 24 hrs following plasmid transfection. Discontinuity in graph indicates a broken Y-axis (H).

We next examined whether TRAF5 may physically and functionally interact with MAVS. In coimmunoprecipitation experiments, TRAF5 bound to MAVS (Figure 3A). However, TRAF5 failed to bind to an inactive deletion mutant of MAVS lacking its TM domain (MAVSΔTM), demonstrating that the TM was important for MAVS to bind to TRAF5, as was previously shown for TRAF3 [25]. TRAF5 binding was restored when the TM domain of MAVS was replaced with the analogous domain from Bcl-xL (MAVS-TM) (Figure 3B), a mutant protein that regains function and binding to TRAF3 also [25], [31]. Mutation of the conserved tryptophan residue in the MAVS CARD (W68A) that is essential for function, also impaired TRAF5 binding (Figure 3B). Thus, the TM domain and CARD are both necessary for MAVS to bind to TRAF5. In addition, as previously shown for TRAF3 [25], we found that TRAF5 associated with MAVS in yeast suggesting a likely direct interaction (Figure 3D).

We previously found that MAVS could interact with TRAF3 and induce its ubiquitination in a CARD-dependent manner. To see if TRAF5 ubiquitination might also be enhanced in response to MAVS activation, we coexpressed TRAF5 with epitope-tagged Ub, along with a constitutively-active form of RIG-I (RIG-IΔRD) or wild-type MAVS. RIG-IΔRD and MAVS both stimulated the ubiquitination of TRAF5 (Figure 3E). In order to examine whether TRAF5 ubiquitination could be stimulated by dimerization of the MAVS CARD we coexpressed MAVS CARD-FPK3 with TRAF3 or TRAF5. In the presence of dimerizer, the self-association of the MAVS CARD through FPK3 was able to induce both TRAF3 and TRAF5 ubiquitination (Figures 3F). Thus, TRAF5, like TRAF3 is a target of the MAVS CARD domain after it is dimerized through the TM domain.

**Figure 4:**
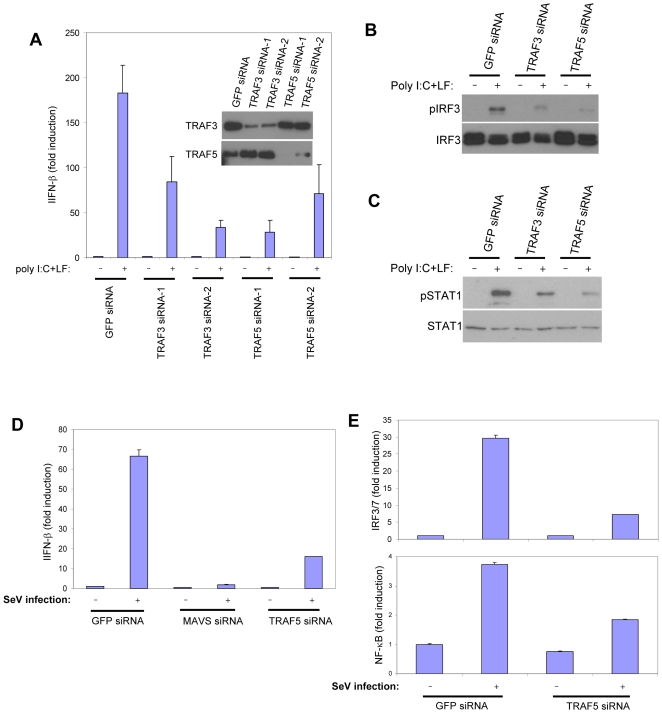
TRAF5 is required for IRF3/7 and NF-κB activation in response to RLR signaling. A HEK293T cells were initially transfected with siRNAs targeting GFP, TRAF3, or TRAF5. 48 hrs later, cells were transfected with pLuc-IFNβ and 6 hrs later, poly I:C was transfected. Luciferase activity was measured 18 hours following poly I:C transfection. In the inset figure, HA-TRAF3 or HA-TRAF5 was cotransfected with siRNAs targeting GFP, TRAF3, or TRAF5 and anti-HA immunoblots were performed on prepared whole cell lysates. B HEK293T cells were initially transfected with siRNAs targeting GFP, TRAF3, or TRAF5. 48 hrs later, cells were transfected with HA-IRF3, lysates were prepared, and anti-HA immunoprecipitates were probed for phosphorylated IRF3 (Ser 396) or total HA-IRF3. Cells were transfected with poly I:C 8 hrs prior to cell lysis. C HEK293T cells were transfected with siRNAs targeting GFP, TRAF3, or TRAF5. Cell lysates probed for phospho-STAT1 (Y701) or total STAT1. Poly I:C was transfected 8 hrs prior to cell lysis. D, E Luciferase reporter assays were performed in HEK293T cells transfected with siRNA targeting GFP, MAVS, or TRAF5. 48 hrs following siRNA transfection, cells were transfected with pLuc-IFN-β, pLuc-PRD(III-I)_3_ (IRF3/7) or pLuc-PRD(II)_2_ (NF-κB). SeV infection was performed 4 hrs following transfection and 20 hrs prior to luciferase assay.

To see whether TRAF5 was required for RIG-I and MAVS-mediated activation of signaling, we knocked down TRAF5 expression using RNA interference (see Materials and Methods and Figure 4A). In luciferase reporter assays, we found that knockdown of TRAF5 expression impaired both IRF3/7 and NF-κB activation downstream by RIG-I and MAVS (Figures 3H and 3I). Thus, like TRAF3, TRAF5 appears to function downstream of MAVS in signal transduction.

### TRAF5 Is Required for the Response to Intracellular RNA

**Figure 5:**
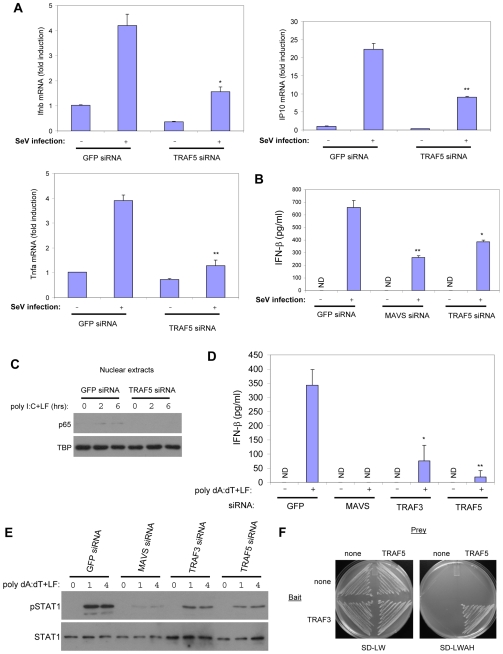
TRAF5 is required for the induction of gene expression downstream of RLR signaling. A HEK293T cells were transfected with siRNAs targeting GFP or TRAF5. 48 hrs later, cells were mock infected or infected with SeV for 8 hrs and total RNA was prepared. *Ifnb*, *IP10*, and *Tnfa* mRNA levels were measured by real-time RT-PCR using specific primers as described in the Materials and Methods section. *GADPH* was used as an internal control. Asterisk, *P*<0.01 versus GFP siRNA + infection (n = 2), double asterisk, *P*<0.005 versus GFP siRNA + infection (n = 2). B, HEK293 cells were transfected with siRNAs targeting GFP, MAVS, or TRAF5. 48 hrs later, cells were mock infected or infected with SeV. Tissue culture supernatants were collected 24 hrs following infection and human IFN-β ELISA was performed. The concentration of IFN-β is shown (mean +/- sd). ND, not detectable. Asterisk, *P* = 0.01 versus GFP siRNA + infection (n = 2), double asterisk, *P*<0.005 versus GFP siRNA + infection (n = 2). C, HEK293 cells were transfected with siRNAs targeting GFP or TRAF5. 48 hrs later, cells were transfected with poly I:C for 2 or 6 hrs or mock treated for 6 hrs. Nuclear extracts were prepared and analyzed for p65 expression by immunoblotting. TBP was used as a loading control. D, HEK293 cells were transfected with siRNAs targeting GFP, MAVS, TRAF3, or TRAF5. 48 hrs later, cells were transfected with poly dA:dT or mock treated. Tissue culture supernatants were collected for human IFN-β ELISA 24 hrs after poly dA:dT transfection. The concentration of IFN-β is shown (mean +/− sd). ND, not detectable. Asterisk, *P*<0.025 versus GFP siRNA + poly dA:dT (n = 2), double asterisk, *P*<0.001 versus GFP siRNA + poly dA:dT (n = 2). E, HEK293 cells were transfected with siRNAs as in (D). 48 hrs later, cells were transfected with poly dA:dT or mock treated for 8 hrs and lysates prepared. Either 1 or 4 ug/ml of poly dA:dT was used as indicated. Lysates were probed for phospho-STAT1 (Y701) or total STAT1. F, Yeast were transformed with the bait or prey constructs indicated as described in the Materials and Methods section and streaked onto minimal SD media lacking leucine and tryptophan (SD-LW) or leucine, tryptophan, adenine, and histidine (SD-LWAH).

To examine whether TRAF5 in fact plays a role in RLR signaling, we tested the effect of the knockdown of TRAF5 expression on signaling activated by transfected poly I:C and viral infection. Knockdown of TRAF5 or TRAF3 by siRNA transfection diminished *Ifnb* promoter induction, IRF3 phosphorylation, and STAT1 phosphorylation in response to transfected poly I:C (Figures 4A, 4B, 4C). TRAF5 was also required for the full induction of the *Ifnb* promoter and activation of IRF3/7 in response to SeV infection (Figures 4D and 4E). Accordingly, TRAF5 knockdown inhibited the upregulation of *Ifnb* and *IP-10* mRNAs in response to SeV infection (Figures 5A). IFN-β production in response to SeV infection was also impaired (Figure 5B). We also examined the effect of TRAF5 knockdown on NF-κB activation by RLR signaling. TRAF5 siRNA transfection inhibited p65/RelA nuclear translocation in response to transfected poly I:C (Figure 4C). Also knockdown of TRAF5 impaired activation of an NF-κB reporter in response to SeV infection (Figure 4E). We also found that the induction of *Tnfa* mRNA in response to SeV infection was also impaired (Figure 5A). Therefore, like TRAF3, TRAF5 is necessary for full IRF3/7 activation and IFNβ induction in response to RLR signaling, whereas TRAF5 is also required for NF-κB activation.

In addition to viral RNA, intracellular dsDNA also induces an innate immune response that promotes IRF3/7 activation. It has recently been shown that transfected poly dA:dT or infection several DNA viruses leads to the activation of the RIG-I-MAVS pathway indirectly via the RNA polymerase-dependent transcription of DNA into dsRNA [14], [15]. To see if TRAF3 and TRAF5 also mediate this pathway, we examined the activation of IFN-β production in cells after transfection with poly dA:dT following the knockdown of TRAF3 or TRAF5 expression. We found that TRAF3 and TRAF5, like MAVS, were both required for IFN-β production and the induction of STAT1 phosphorylation in response to transfected poly dA:dT (Figures 5D and 5E). Thus, the induction of IFN-β by intracellular B-DNA, like RNA is dependent on TRAF3 and TRAF5.

Our data together suggest that TRAF3 and TRAF5 each have at least some nonredundant essential functions in the activation of IRF3/7 signaling downstream of MAVS. One possibility is that TRAF3 and TRAF5 can function together in a complex to signal. To test this possibility we examined whether these two proteins might be able to associate with one another in a yeast two-hybrid interaction assay. We were able to detect a TRAF3-TRAF5 interaction in yeast, a result consistent with the possibility that these two ubiquitin ligases may function as a heterodimeric complex in RLR signaling (Figure 5F).

### NEMO Is Recruited to MAVS in a TRAF3/5-Dependent Manner

**Figure 6:**
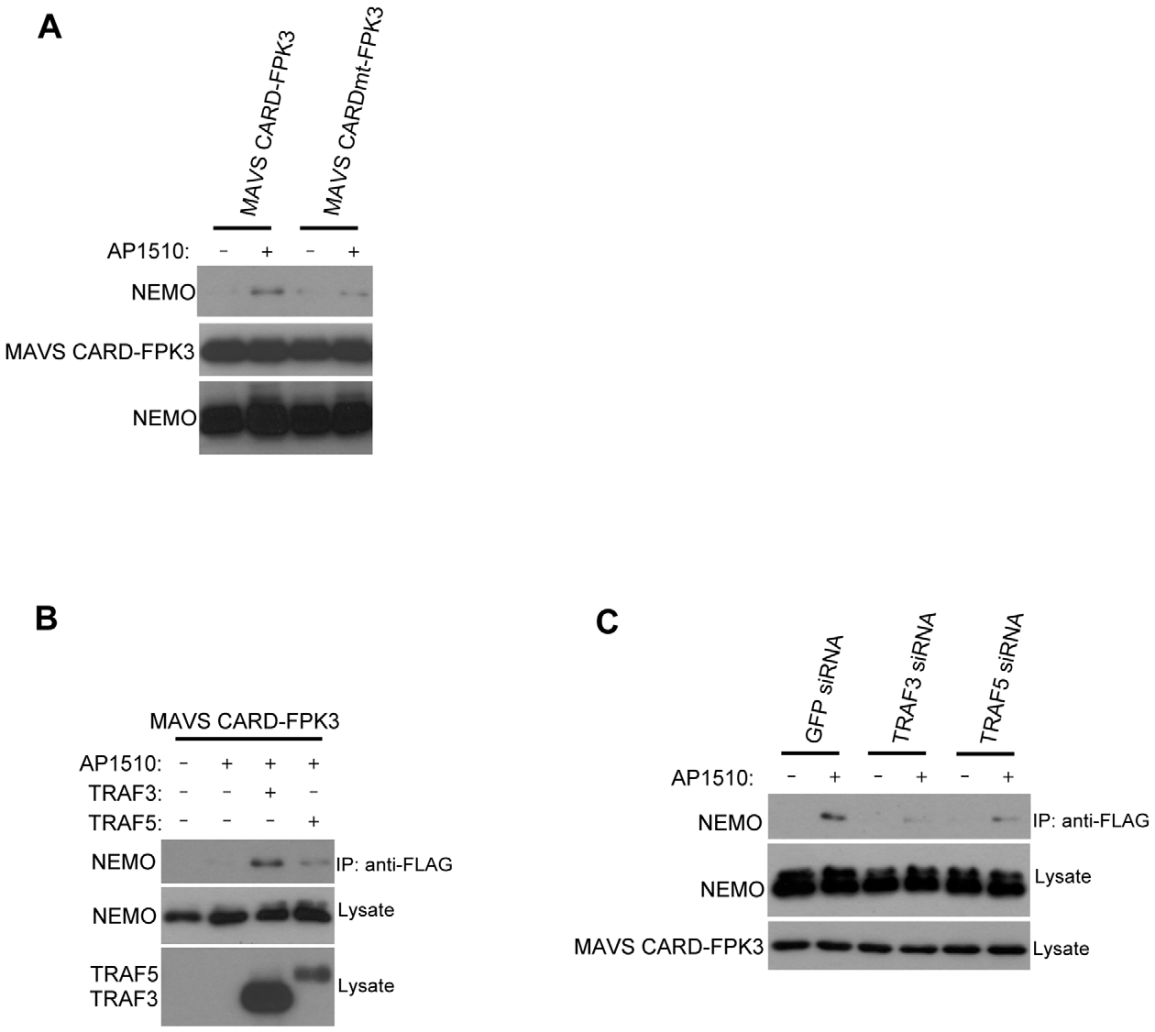
NEMO is recruited to the MAVS CARD in a TRAF3/5-dependent manner. A HEK293T cells were transfected with AU1-NEMO and FLAG-MAVS CARD-FPK3 or FLAG-MAVS CARDmt-FPK3 where indicated. MAVS CARDmt contains a substitution of alanine for tryptophan at residue 68. Lysates were prepared and anti-FLAG immunoprecipitates were probed for AU1-NEMO. Lysates were probed for FLAG-MAVS-CARD-FPK3 protein and AU1-NEMO. AP1510 was added to media 12 hrs prior to cell lysis. B HEK293T cells were transfected with AU1-NEMO and FLAG-MAVS CARD-FPK3, HA-TRAF3 or HA-TRAF5 where indicated. Anti-FLAG-immunoprecipitates were probed for AU1-NEMO. Lysates were immunoblotted for FLAG-MAVS proteins and AU1-NEMO. AP1510 was added to media 12 hrs prior to cell lysis. C HEK293T cells first transfected with siRNAs targeting GFP, TRAF3, or TRAF5. 48 hrs later, cells were transfected with FLAG-MAVS CARD-FPK3 and AU1-NEMO. Anti-FLAG-immunoprecipitates were probed for AU1-NEMO. Lysates were immunoblotted for FLAG-MAVS proteins and AU1-NEMO. AP1510 was added to media 12 hrs prior to cell lysis.

Recently NEMO was identified as an essential adaptor molecule required for IRF3 and IRF7 activation downstream of MAVS in RLR signaling pathways [24]. NEMO was previously suggested to mediate the recruitment of TANK and TBK1 to the MAVS signaling complex although any physical association with NEMO and MAVS has yet to be demonstrated. We first examined whether NEMO could be recruited to the MAVS signaling complex. We tested if the MAVS CARD, when oligomerized and activated through FPK3, could coprecipitate NEMO from cells. We found that NEMO associated with the MAVS CARD-FPK3 fusion when it was oligomerized, but not with the mutant form (Figure 6A). To check if this interaction was influenced by TRAF3 or TRAF5, we examined the effect of cotransfecting an additional expression plasmid for TRAF3 or TRAF5. Additional TRAF3 or TRAF5 expression resulted in an enhancement in the association of NEMO with MAVS CARD-FPK3 (Figure 6B). Conversely, we found that association of NEMO with MAVS CARD-FPK3 was diminished when the expression of TRAF3 or TRAF5 was knocked down by RNAi (Figure 6C). Thus, TRAF3 and TRAF5 both mediate the recruitment of NEMO to the MAVS signaling complex.

## Discussion

Intracellular RNA is recognized by the RNA helicases RIG-I and MDA-5 and leads to self-association of MAVS on mitochondria and the activation of pathways leading to IRF3/7 and NF-κB activation. Previously, we found that dimerization of MAVS results in its ability to associate with and activate the downstream molecule TRAF3 [25]. This interaction is dependent on the N-terminal CARD domain of MAVS. However, genetic disruption of TRAF3 has been shown to only partially inhibit the response to virus infection [18], [28]. Also TRAF3 has been shown to not be required for NF-κB activation [18]. Our data in this report find that the related molecule TRAF5 plays an important role in both IRF3/7 and NF-κB activation downstream of MAVS. The N-terminal CARD of MAVS directly associates with and activates TRAF5. TRAF3 and TRAF5 both appear to be required for the recruitment of NEMO to the MAVS CARD. In addition to the response to cytosolic RNA, TRAF3 and TRAF5 also play a role in production of IFN-β in the response to cytosolic B-DNA.

The identification of TRAF5, in addition to TRAF3, as a downstream target of MAVS may explain the partial defects in IRF3 activation and IFN-β induction by RNA virus infection observed in TRAF3 −/− MEFs [18], [32]. Our results suggest that TRAF3 and TRAF5 each have at least some nonredundant functions in IRF3 activation while TRAF5 has an additional role in NF-κB activation. How TRAF3 and TRAF5 mediate the recruitment of NEMO remains to be determined. Given the ability of MAVS to induce the ubiquitination of both TRAF3 and TRAF5 in a CARD-dependent manner, it is possible that TRAF3 and TRAF5 autoubiquitination may lead to their recognition by NEMO through its previously characterized two ubiquitin binding domains, located in the CC2-LZ region and C-terminal zinc finger [33], [34]. In fact, previous studies demonstrated that mutations in either of these two ubiquitin binding domains in NEMO leads to an impairment in the activation of type I interferon production by viral infection or overexpressed MAVS [24], [32]. Also, it has been shown recently that the *in vitro* ubiquitination of TRAF3 allows it to bind to NEMO [32]. We propose that autoubiquitination of TRAF3 and TRAF5 in response to MAVS dimerization leads to the recruitment of NEMO via its ubiquitin binding domains to the MAVS signaling complex (see Figure 6).

**Figure 7:**
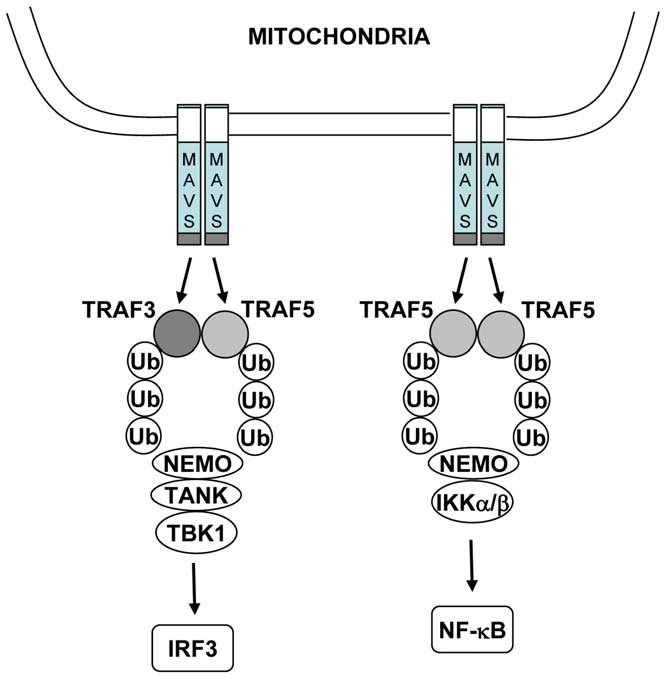
Schematic representation of model for signal transduction downstream of MAVS. MAVS homodimers, induced by binding of the activated forms of the RLRs RIG-I and MDA-5, are able to signal downstream in a TRAF3 and TRAF5-dependent manner to activate IRF3/7 and in a TRAF5-dependent manner to activate NF-κB. NEMO may be recruited to the MAVS signaling complex by ubiquitinated TRAF3 and TRAF5, and may lead to IRF3 phosphorylation through the recruitment of TANK and TBK1. Alternatively, NEMO may be recruited to a distinct MAVS signaling complex by ubiquitinated TRAF5, thereby promoting the recruitment IKKα and IKKβ, and leading to NF-κB activation. The mechanisms underlying the selective recruitment of signaling molecules to the MAVS signaling apparatus remain to be determined.

Previously, TRAF3 knockout cells were found to display a partial defect in type I interferon production in response to RNA virus infection, but an enhanced activation of NF-κB activation and production of inflammatory cytokines as a result of NIK stabilization. Our results clarify how NF-κB is activated downstream of MAVS by identifying TRAF5 as a mediator of the activation of NF-κB in addition to IRF3. The role of the TRAF family member TRAF6 has been previously implicated to be necessary for full NF-κB activation in response to viral infection and cytosolic RNA [35]. We have found that mutation of the two TRAF6 binding motifs in MAVS has little effect on its ability to activate NF-κB (Figure 1D). Also, unlike TRAF3 and TRAF5, we have been unable to detect binding of TRAF6 to the MAVS CARD (data not shown). It is possible that full activation of NF-κB may require both CARD-dependent TRAF5-dependent and CARD-independent TRAF6-dependent interactions. Also there may be differences in the use of signaling molecules between murine and human cells. Of note, murine and human TRAF6 have been shown to display significant functional differences in the induction of apoptosis and NF-κB [36]. It remains to be determined how TRAF5 but not TRAF3 selectively mediates NF-κB activation downstream of MAVS. While TRAF3 and TRAF5 may act coordinately to recruit NEMO and TANK to activate IRF3, TRAF5 alone may recruit NEMO and IKK kinase subunits to activate NF-κB (Figure 7). It remains to be investigated how such differential recruitment and/or activation may occur.

Our results reveal a role for TRAF5 in signaling downstream of MAVS. While TRAF3 and TRAF5 are both important for IRF3/7 activation and type I interferon production, TRAF5 also plays a role in NF-κB activation and proinflammatory cytokine induction. A further understanding of the molecular mechanism of MAVS signal transduction through TRAF3 and TRAF5 will be important for the development of novel anti-viral therapeutic approaches.

## Materials and Methods

### Cell Culture and Transfections

HEK293 and HEK 293T cells were obtained from ATCC. All cells were grown in Dulbecco's modified Eagle's medium supplemented with 10% fetal bovine serum, 50 units penicillin/ml, and 50 ug streptomycin/ml (Invitrogen). Transient transfections in HEK293T cells and MEFs were performed using LipofectAMINE 2000 according to manufacturer's instructions (Invitrogen). Poly I:C was transfected at 4 ug/ml with LipofectAMINE 2000. For all luciferase assays, cells were cotransfected with pRL-TK *Renilla* luciferase (Promega) and luciferase activity was measured by a dual-luciferase reporter assay system (Promega) using *Renilla* luciferase as an internal control. Cells were lysed and measurements were taken 20–24 hrs following plasmid transfection.

### Reagents

Poly I:C and poly dA:dT was purchased from GE Healthcare Life Sciences. bbit polyclonal antibody to MAVS was purchased from Bethyl Laboratories. Mouse monoclonal HA and rabbit polyclonal HA and AU1 antibodies were from Covance. p65 mouse monoclonal antibody (F-6) was purchased from Santa Cruz. FLAG M2 antibody was purchased from Sigma. PhosphoIRF3 (Ser 396), phosphoIκBα (Ser 32), and phosphoSTAT1 (Tyr 701) rabbit polyclonal antibodies were from Cell Signaling. Mouse monoclonal antibody recognizing TBP was obtained from Abcam. AP1510 was obtained from ARIAD and used at 100 nM.

### Plasmids

Expression construct for FLAG- and HA-tagged TRAF5 were constructed by PCR amplification and standard recombinant DNA techniques. TRAF3ΔN and TRAF5ΔN contain N-terminal deletions of the first 97 from TRAF3 and the first 90 codons from TRAF5, respectively. The expression construct for HA-IkBα has been described previously [37]. RIG-IΔRD is a C-terminal deletion mutant of RIG-I containing codons 1 to 734, which has been previously shown function as a constitutively active mutant [38]. AU1-tagged NEMO was constructed by ligating the cDNA fragment from pcDNA3-HA-NEMO [39]into pcDNA3-AU1. Authenticity of all constructs were confirmed by sequencing (UCLA sequencing core). Point mutations and deletion mutants were constructed by PCR mutagenesis (Stratagene). AU1-Ub expression construct has been described previously [39]. MAVS EE/DD contains substitutions of aspartic acid for glutamic acid at codons 155 and 457 located in two consensus TRAF6 binding motifs [35]. Expression constructs for HA-TRAF3, MAVS WT, MAVS W68A, MAVS Q145N, MAVSΔTM, MAVS-TM, MAVSΔTMx2, MAVS CARD, MAVS-CARD-FPK3, MAVS CARDmt-FPK3 have been described previously [25]. pLUC-IFNβ, pLUC-PRD(III-I)_3_, and pLUC-PRD(II)_2_ have been described previously [40].

### Immunoblotting and Immunoprecipitations

For all coimmunoprecipitation assays, HEK293T cells were washed once with phosphate-buffered saline (PBS) (Invitrogen) and then lysed in EBC150 lysis buffer (50 mM Tris HCl pH 8.0, 150 mM NaCl, 0.5% Nonidet P-40, 50 mM NaF, 0.1 mM Na_3_VO_4_, 1 mM DTT). Lysis buffer was supplemented with Complete EDTA-free protease inhibitor cocktail (Roche). Ubiquitination of immunoprecipitated TRAF3 and TRAF5 was performed as described previously [39]. Nuclear extracts were prepared as described previously [41].

### RNA Interference

The target sequences for the siRNAs obtained from Dharmacon used in this study are as follows (sense strand): GFP, GCAGAAGAACGGCAUCAAG; MAVS, AAGUAUAUCUGCCGCAAUU; TRAF3#1, AGAGUCAGGUUCCGAUGAU; TRAF3#2, GCAAGUGGCUCGGAACACA; TRAF5#1, GGUCACACCUGUCCCUAUA; TRAF5#2, GGAUGUAAUGCCAAGGUUA; TANK, GGAUAGAGAUUCUGCAGUA. For TRAF3 and TRAF5 knockdown, siRNAs corresponding to TRAF3#1 and TRAF5#1 target sites were used unless otherwise indicated.

### Viral Infections and Nucleic Acid Stimulation

For infections with Sendai virus (Cantell strain; Charles River laboratories), HEK293T or HEK293 cells were infected for 20 hours at 400 hemagglutinating (HA) units/ml culture media. In brief, cells seeded on 12 well tissue culture plates were washed once with PBS, and then incubated with Sendai Virus diluted in 0.5 ml serum-free DMEM. After one hour, 0.5 ml of DMEM supplemented with 20% fetal bovine serum, 50 units penicillin/ml, and 50 µg streptomycin/ml was added. For experiments in which nucleic acid stimulation was performed, either poly I:C or poly dA:dT (4 ug/ml) was complexed with LipofectAMINE 2000 (4 ul/ml) (Invitrogen) for 15 min prior to addition to media.

### Measurement of IFN-β

Human IFN-β ELISA were performed according to the manufacturers instructions (PBL). 48 hrs following the first siRNA transfection, cells were infected with SeV and supernatants were collected 24 hrs later.

### RT-PCR

Total RNA was isolated with Trizol reagent (Invitrogen) and cDNA was reverse-transcribed with oligo-dT primer using SuperScript III reverse transcriptase (Invitrogen) according to the manufacturer's instructions. Real-time PCR analysis was performed using the iQ SYBR Green Supermix (BioRad) on an iCycler iQ real-time PCR detection system (BioRad). PCR was performed with the following primers: *Ifnb*, 5′- CAGCAATTTTCAGTGTCAGAAGCT-3′ and 5′-TCATCCTGTCCTTGAGGCAGTAT-3′; *IP10*, 5′-TGACTCTAAGTGGCATTCAAGG-3′ and 5′-GATTCAGACATCTCTTCTCACCC-3′; *Tnfa*, 5′-CAGAGGGAAGAGTCCCCCAGGGACC-3′ and 5′-CCTTGGTCTGGTAGGAGACGGCGATG-3′; *GADPH*, 5′- CTGGGCTACACTGAGCACCAG-3′ and 5′- CCAGCGTCAAAGGTGGAG-3′.

### RNA Interference

HEK293 and HEK293T cells were transfected with 100 nM 21mer siRNA oligonucleotide using LipofectAMINE 2000 (Invitrogen) according to manufacturer's instructions. 24 hrs later, cells were transfected once more. Viral infections, plasmid and poly I:C transfections were performed the next day.

### Yeast Two-Hybrid Interactions

MAVS, TRAF3, and TRAF5 cDNAs were cloned into the pGBKT7 bait and pGADT7 prey vectors (Clontech). Constructs were transformed into AH109 yeast (Clontech) by the lithium acetate method and multiple colonies grown on minimal media lacking leucine and tryptophan were streaked out onto minimal media lacking leucine and tryptophan or leucine, tryptophan, adenine, and histidine (Clontech). Plates were incubated at 30°C overnight until colonies formed.

### Statistical Analysis

Statistical significance of any differences in mRNA levels or cytokine concentration was determined by Student's one-sided *t*-test.
